# HPV-transformed cells exhibit altered HMGB1-TLR4/MyD88-SARM1 signaling axis

**DOI:** 10.1038/s41598-018-21416-8

**Published:** 2018-02-22

**Authors:** Mirian Galliote Morale, Walason da Silva Abjaude, Aline Montenegro Silva, Luisa Lina Villa, Enrique Boccardo

**Affiliations:** 1 Department of Biochemistry, Institute of Chemistry, Universidade de São Paulo, São Paulo, Brazil; 2 Centre of Translational Oncology, Instituto do Câncer do Estado de São Paulo (ICESP), São Paulo, Brazil; 3 Department of Radiology and Oncology, Faculdade de Medicina, Universidade de São Paulo, São Paulo, Brazil; 4 Department of Microbiology, Institute of Biomedical Sciences, Universidade de São Paulo, São Paulo, Brazil

## Abstract

Cervical cancer is one of the leading causes of cancer death in women worldwide. Persistent infection with high-risk human papillomavirus (HPV) types is the main risk factor for the development of cervical cancer precursor lesions. HPV persistence and tumor development is usually characterized by innate immune system evasion. Alterations in Toll-like receptors (TLR) expression and activation may be important for the control of HPV infections and could play a role in the progression of lesions and tumors. In the present study, we analyzed the mRNA expression of 84 genes involved in TLR signaling pathways. We observed that 80% of the differentially expressed genes were downregulated in cervical cancer cell lines relative to normal keratinocytes. Major alterations were detected in genes coding for several proteins of the TLR signaling axis, including TLR adaptor molecules and genes associated with MAPK pathway, NFκB activation and antiviral immune response. In particular, we observed major alterations in the HMGB1-TLR4 signaling axis. Functional analysis also showed that HMGB1 expression is important for the proliferative and tumorigenic potential of cervical cancer cell lines. Taken together, these data indicate that alterations in TLR signaling pathways may play a role in the oncogenic potential of cells expressing HPV oncogenes.

## Introduction

Cervical cancer is one of the leading causes of cancer death in women worldwide. Persistent infection with high-risk human papillomavirus (HPV) types is the main risk factor for the development of cervical cancer precursor lesions. The majority of infected women effectively eliminate the virus and only a minority develops cancer. In some cases HPV-induced innate and adaptive immune responses are unable to eliminate the virus leading to persistent infection increasing the likelihood of cervical intraepithelial neoplasia (CIN) and cancer development^1^.

Two HPV oncoproteins, E6 and E7, are the only viral products constitutively expressed in cervical tumors and are required for the maintenance of the transformed phenotype^2^. These proteins are responsible for alterations in several signaling pathways of the host cell, including those involved in regulating cell differentiation, proliferation, and apoptosis^3^. Both E6 and E7 are also involved in the deregulation of the immune system and the inflammatory process. Several pathways are affected by HPV infection, including inhibition of interferon responses by E6 and E7 via interaction with interferon regulatory factors (IRFs) 1, 2 and 3; inhibition of immune system via downregulation of proinflammatory cytokines such as interleukin 6 (IL6); and modulation of innate immunity via alterations in Toll-like receptors (TLR) expression^4,5^.

The innate immune system is the first line of cells defense against pathogens. TLR are a family of membrane proteins that actively participate in this process. These receptors bind to molecular patterns such as lipopolysaccharide (LPS), double-stranded RNA (dsRNA), and flagellin from many pathogens including bacteria, fungi, and viruses, as well as, molecular patterns coming from danger signals produced by cells on distress. This interaction triggers a signaling cascade, starting with recruitment of adaptor molecules followed by activation of transcription factors and production of proinflammatory cytokines, which ultimately can eliminate the infectious agent^6^.

Immune system evasion can lead to HPV persistence and tumor development. Therefore, alterations in TLR expression and activation may be important for control of HPV infections and progression of HPV-associated lesions and cancers^7^. HPV16 clearance in naturally infected individuals has been shown to be associated with increased expression of TLR2, TLR3, TLR7, TLR8, and TLR9^8^. Conversely, a positive correlation has been detected between the expression of TLR4, TLR7, and TLR9 and the development and progression of CIN and cervical carcinoma associated with HPV16^9^.

The alterations in the expression and function of TLR pathway molecules in cells expressing HPV genes have not been investigated in depth. In this study, we analyzed the expression of 84 genes involved in TLR signaling pathways, and observed that several of these genes were differentially expressed in HPV-positive cervical cancer cells when compared to normal cells. Importantly, 80% of the genes analyzed were downregulated in HPV-positive cervical cancer cell lines relative to normal keratinocytes. Major alterations were detected in genes coding for proteins of the TLR4 signaling axis, including the adaptor molecules MyD88 (myeloid differentiation primary response 88) and SARM1 (sterile alpha and TIR motif containing 1), the NFκB activation complex components, Ube2N (ubiquitin conjugating enzyme E2 N) and TRAF6 (TNF receptor associated factor 6), and the effector protein HMGB1 (high mobility group box 1). Functional analysis showed that HMGB1, a protein related to stimulation of inflammation after TLR activation^10^, was elevated in cells expressing HPV oncogenes. In addition, HMGB1 silencing greatly affected the proliferative and tumorigenic potential of HPV-positive tumor cells. Taken together, these results indicate that alterations in the TLR signaling pathway may affect the oncogenic potential of cells expressing HPV genes.

## Results

### Cervical cancer-derived cell lines exhibit major alterations in the expression of TLR-related genes

To determine the overall effect of HPV on the TLR pathway, we analyzed the mRNA expression of 84 genes involved in TLR signaling in normal primary human keratinocytes (PHK) and the cervical cancer cell lines C33A, HeLa, and SiHa using a commercial quantitative PCR array. The analyses were conducted using a two-step approach; first, we compared the gene expression profile of all tumor cell lines using PHK as a reference group. In a second approach, in order to evaluate the effect of HPV presence on the expression of these genes we compared the expression profiles of the HPV-positive cell lines HeLa (HPV18) and SiHa (HPV16) with the observed in HPV-negative cell line C33A. The results of these analyses are presented in Supplementary Tables SI and SII.

Using PHK as reference, nine of the 84 genes analyzed were differentially expressed (p-value ≤ 0.05) exclusively in HeLa, whereas only TLR1 was differentially expressed exclusively in SiHa cells (Table 1 Section A). In addition, six genes were differentially expressed between both HPV-positive cell lines and PHK. Of these genes only TLR4 was upregulated (Table 1 Section B). In addition, 12 genes were differentially expressed in the three cervical cancer cells lines when compared to PHK (Table 1 Section B). SARM1 was the only gene which mRNA levels were upregulated in all three cells lines C33A, HeLa and SiHa cells. (Table 1 Section C).

**Table 1:** Genes from TLR-regulated pathway differentially expressed between cervical cancer cell lines and normal keratinocytes (>2-fold or <−2-fold change and p value ≤ 0.05). NS: non-significant.

*Symbol*	Description	*HeLa*		*SiHa*		*C33A*	
		Fold regulation	p value	Fold regulation	p value	Fold regulation	p value
***A***	**Genes differentially expressed exclusively in HeLa or SiHa**						
*CCL2*	Chemokine (C-C motif) ligand 2	16.374	0.000	NS	NS	NS	NS
*CD180*	CD180 molecule	6.409	0.046	NS	NS	NS	NS
*EIF2AK2*	Eukaryotic translation initiation factor 2-alpha kinase 2	−7.835	0.03	NS	NS	NS	NS
*IKBKB*	Inhibitor of kappa light polypeptide gene enhancer in B-cells, kinase beta	−2.651	0.005	NS	NS	NS	NS
*MAP3K1*	Mitogen-activated protein kinase kinase kinase 1	−2.822	0.000	NS	NS	NS	NS
*PRKRA*	Protein kinase, interferon-inducible double stranded RNA dependent activator	−2.373	0.017	NS	NS	NS	NS
*TICAM2*	Toll-like receptor adaptor molecule 2	−3.411	0.033	NS	NS	NS	NS
*TLR1*	Toll-like receptor 1	NS	NS	−16,329	0,043	NS	NS
*TLR9*	Toll-like receptor 9	−5.303	0.047	NS	NS	NS	NS
*TRAF6*	TNF receptor-associated factor 6	−6.007	0.029	NS	NS	NS	NS
***B***	**Genes differentially expressed in HeLa and SiHa**						
*MAP3K7*	Mitogen-activated protein kinase kinase kinase 7	−3.182	0.004	−2.197	0.005	NS	NS
*MAP4K4*	Mitogen-activated protein kinase kinase kinase kinase 4	−4.902	0.005	−2.732	0.009	NS	NS
*REL*	V-rel reticuloendotheliosis viral oncogene homolog (avian)	−4.287	0.001	−2.989	0.002	NS	NS
*TICAM1*	Toll-like receptor adaptor molecule 1	−5.04	0.002	−5.637	0.001	NS	NS
*TLR10*	Toll-like receptor 10	−8.694	0.002	−4.741	0.005	NS	NS
*TLR4*	Toll-like receptor 4	38.497	0.000	8.864	0.039	NS	NS
***C***	**Genes differentially expressed in HeLa, SiHa and C33A**						
*IL12A*	Interleukin 12A (natural killer cell stimulatory factor 1)	−5.205	0.001	−2.65	0.002	3,725	0,000
*IL1A*	Interleukin 1, alpha	−820.296	0.003	−478.054	0.003	−2725,868	0,002
*IL1B*	Interleukin 1, beta	−2407.522	0.006	−187.375	0.006	−2584,793	0,005
*IRAK4*	Interleukin-1 receptor-associated kinase 4	−5.897	0.01	−2.99	0.046	4,039	0,000
*LY96*	Lymphocyte antigen 96	−739.292	0.032	−99.892	0.033	−18,885	0,038
*PELI1*	Pellino homolog 1 (*Drosophila*)	−10.954	0.004	−6.118	0.005	−2,967	0,013
*PTGS2*	Prostaglandin-endoperoxide synthase 2	−174.047	0.000	−19.096	0.001	−1356,650	0,000
*RIPK2*	Receptor-interacting serine-threonine kinase 2	−5.439	0.002	−2.578	0.004	2,441	0,000
*SARM1*	Sterile alpha and TIR motif containing 1	6.948	0.004	116.056	0.022	18,262	0,000
*TLR2*	Toll-like receptor 2	−560.278	0.006	−55.568	0.006	−77,127	0,006
*TLR3*	Toll-like receptor 3	−12.409	0.002	−4.412	0.005	−2,107	0,025
*TLR5*	Toll-like receptor 5	−27.538	0.011	−11.953	0.013	−3,107	0,045

**Figure 1:**
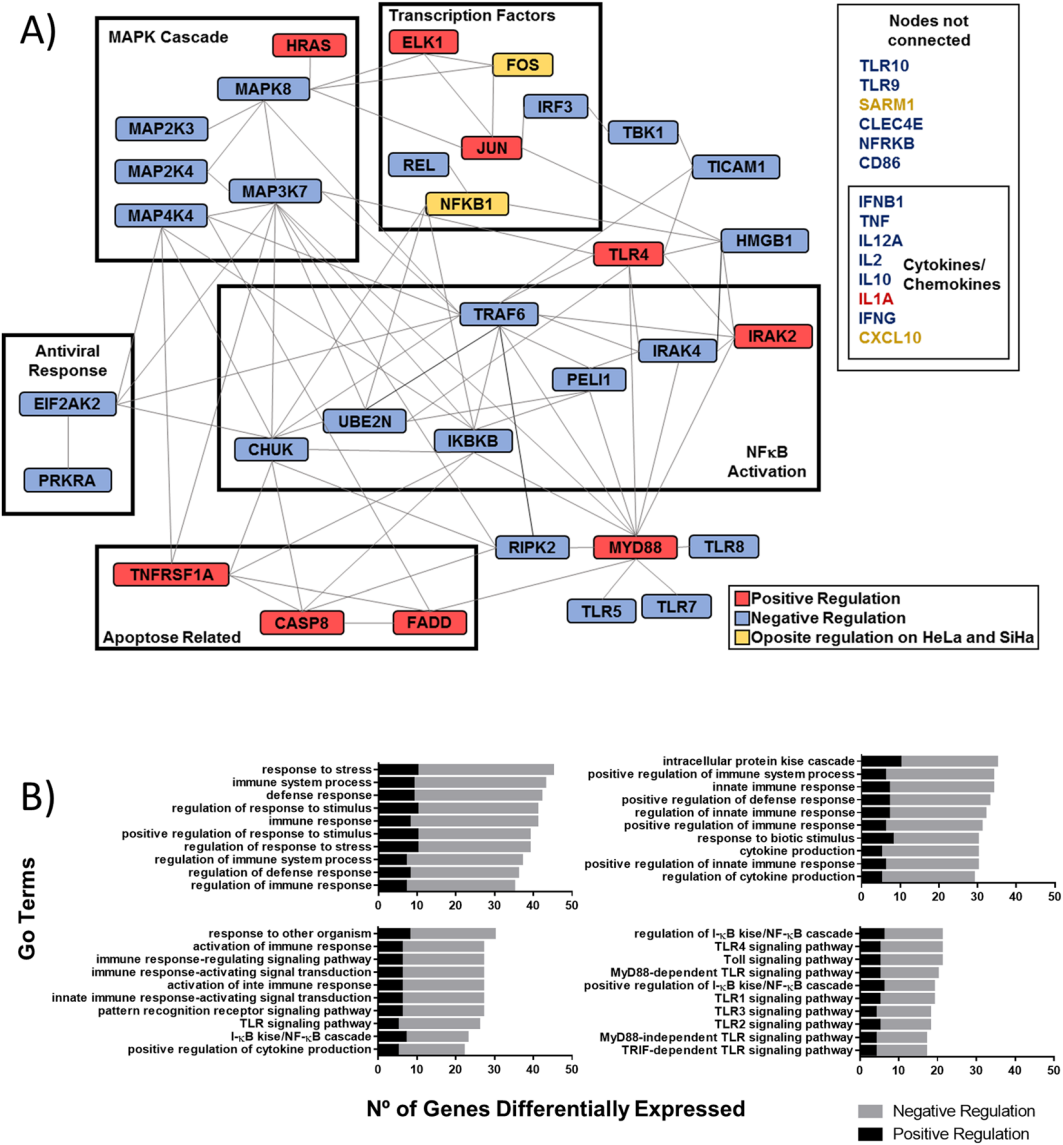
Cervical cancer cell lines exhibit altered mRNA expression of genes involved TLR-signaling pathway. (**A**) Pathway network analysis of 47 TLR pathway genes differentially expressed in HPV positive tumor cell lines (HeLa and SiHa) relative to HPV negative tumor cells (C33A). Genes clustered by protein function and delimited by boxes (>2-fold or < −2-fold change and p value ≤ 0.05). Genes upregulated are red colored, genes downregulated are blue colored, and yellow-colored genes had opposite results in HeLa and SiHa cell lines (STRING: functional protein association networks, string-db.org/). (**B**) Biological processes distribution of genes differentially expressed in HPV positive tumor cell lines relative to HPV negative tumor cells. (WEB-based GEne SeT AnaLysis Toolkit, www.webgestalt.org/).

In total, 47 genes were differentially expressed (p value ≤ 0.05) between HPV positive cell lines, HeLa and SiHa, when compared to HPV negative cell line, C33A. A functional protein association network of these genes highlighting clusters related to specific functions was generated (Fig. 1A). Of the 47 genes, approximately 80% were downregulated in HeLa and SiHa cells relative to the C33A cell line, including most genes related to MAPK signaling, NFκB activation, antiviral response, and cytokine and chemokine production. Conversely, some genes including apoptosis mediators TNFRSF1A, caspase 8, and FADD, adaptor molecule MyD88, and TLR4 were upregulated. Forty enriched Gene Ontology categories were identified (P < 0.01) (Fig. 1B). Interestingly, most categories were associated with general regulation of innate immunity and not specifically with regulation of TLR7, TLR8, and TLR9, which are responsible for triggering antiviral response.

### Validation of differentially expressed genes

Based on the results of the expression analysis presented and data from the literature, the following genes were selected for validation: MyD88, SARM1, Ube2N, IRAK4 (interleukin 1 receptor associated kinase 4), HMGB1, TRAF6, and TLR4.

**Figure 2:**
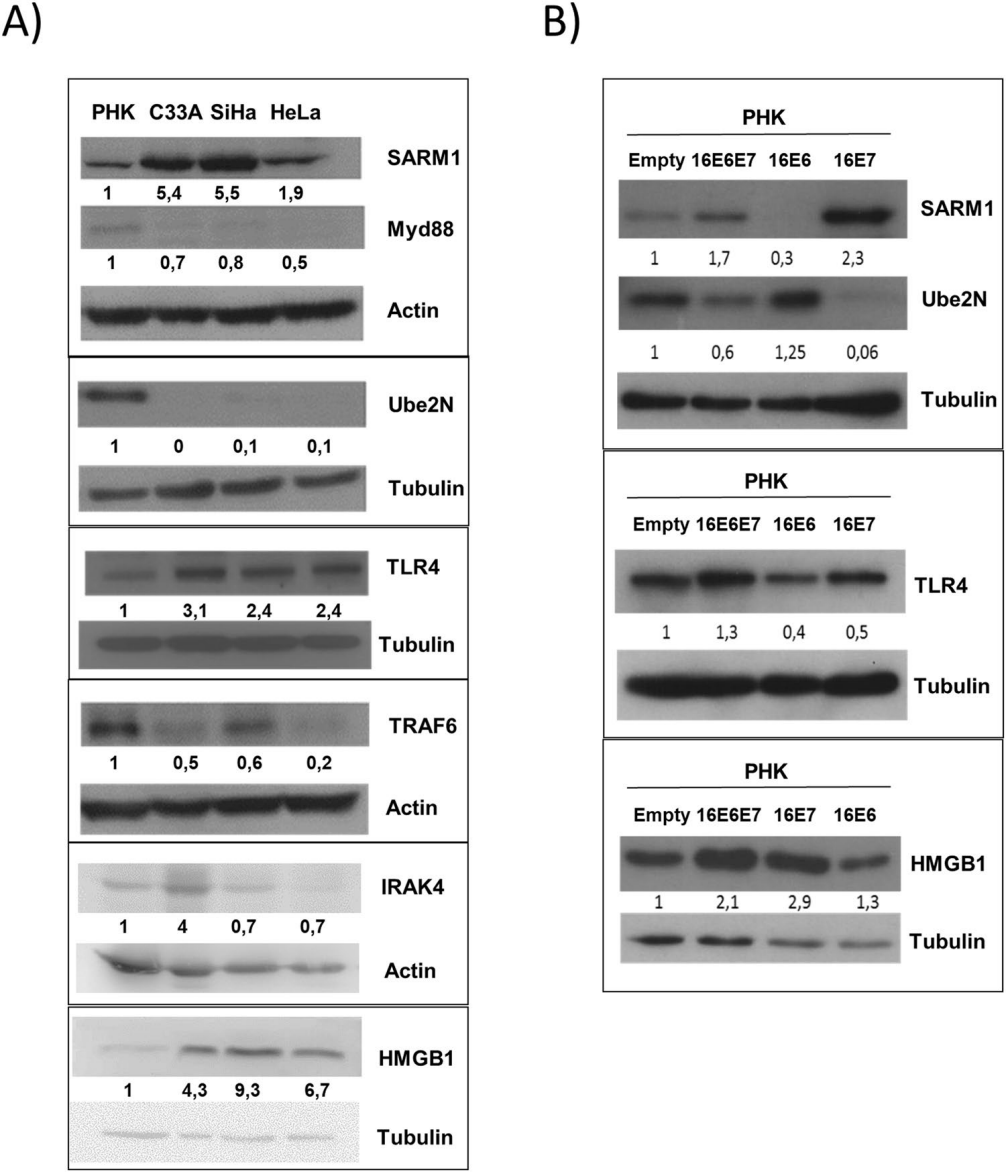
Cervical cancer cell lines and PHK expressing HPV16 oncogenes exhibit alterations in the expression of TLRs related proteins. (**A**) Tumor-derived cell lines C33A, SiHa, and HeLa, (**B**) PHK expressing HPV16 oncogenes E6 and/or E7. Protein levels were determined by Western blot analysis from cell culture extracts. Signals obtained were quantified using ImageJ software and are reported as expression relative to normal keratinocytes (PHK). Full-length blots are presented in a Supplementary Figure.

The levels of these proteins were determined by western blot in total protein extracts from PHK and C33A, SiHa, and HeLa tumor cell lines. Our results confirmed changes in the expression of several proteins. First, we observed that the adaptor molecule MyD88 was downregulated whereas SARM1 and TLR4 were upregulated in all three cervical cancer cell lines relative to normal keratinocytes (Fig. 2A). Besides, TRAF6 and Ube2N protein levels were notably lower in tumor cell lines than in PHK (Fig. 2A) confirming the results from gene expression analysis for HPV positive cell lines. Of note, tumor cell lines exhibited higher HMGB1 protein levels despite having lower levels of mRNA. This observation suggests that in our model HMGB1 protein levels may be upregulated by post-transcriptional mechanisms (Fig. 2A and Supplementary Table II).

### HPV16 E7 alters the expression of HMGB1, SARM1, TLR4 and Ube2N

Considering the results described above we investigated the participation of HPV 16 E6 and E7 in the differential expression of proteins involved in TLR signaling pathways. For this purpose, we determined the levels of some of these proteins in PHK transduced with retroviral vectors expressing HPV16 E6 and/or E7.

Adaptor protein SARM1, TLR4 and HMGB1 showed higher expression levels in the PHK expressing E7 or E6/E7 when compared to PHK transduced with an empty vector or a vector expressing only HPV16 E6 (Fig. 2B). On the other hand, cells expressing E7 or E6/E7 exhibited reduced Ube2N expression when compared to control PHK or cells expressing only E6 (Fig. 2B). These results are in agreement with those obtained using cervical cancer-derived cell lines (Fig. 2A) and strongly suggest the involvement of HPV16 E7 in the deregulation of these factors expression. Interestingly, despite the fact that C33A is not transformed by HPV, this cell line exhibit higher protein levels of HMGB1 than normal PHK. Considering that C33A has alterations on p53 and pRb^11^, the same proteins E6 and E7 of HPV interact with, this is an indication of a common pathway of HMGB1 regulation on cervical cancer cells.

### HMGB1 expression is upregulated in organotypic cultures expressing HPV16 E6E7 and SCC samples

**Figure 3:**
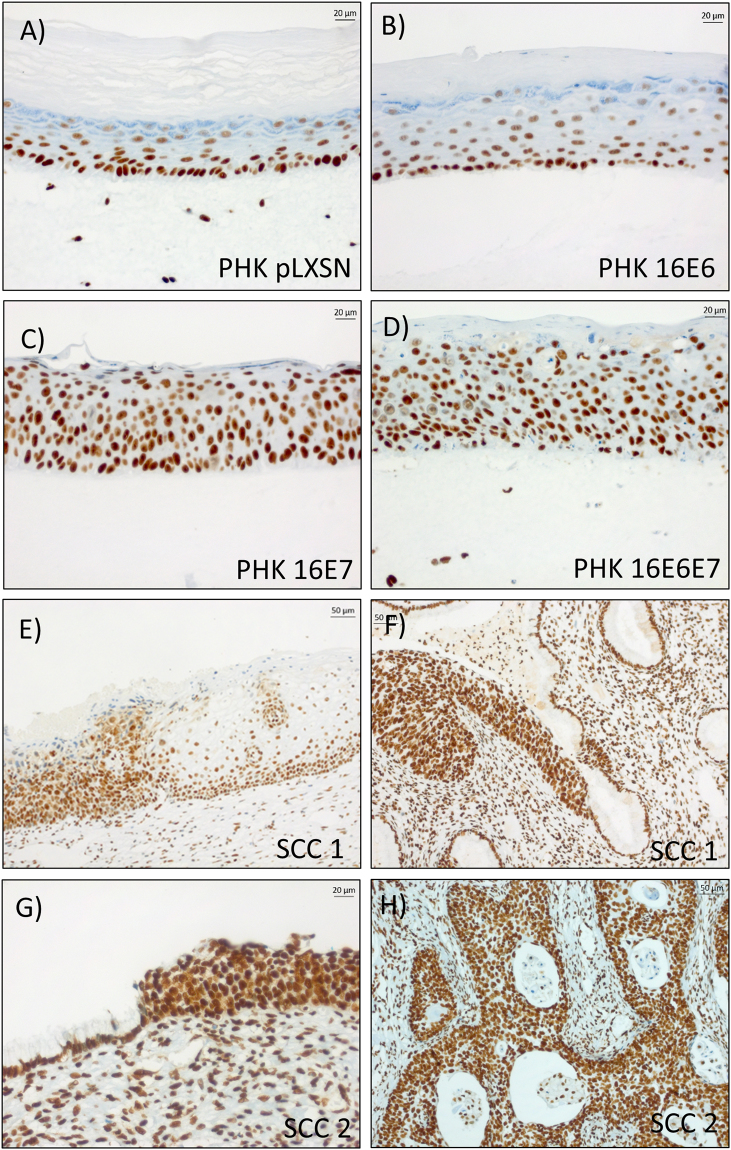
HMGB1 is upregulated in epithelial rafts expressing HPV16 E7 and in squamous cell carcinomas. The expression of HMGB1 was determined by immunohistochemistry in organotypic cultures sections of (**A**) control; (**B**) HPV16 E6 expressing; (**C**) HPV16 E7 expressing and (**D**) with HPV16 E6E7 expressing keratinocytes and in squamous cell carcinomas (**E**,**F**) sample 1 and (**G**,**H**) sample 2. Note limit area between normal epithelium and high-grade lesion (**E**,**G**).

To investigate the effect of HPV16 E6E7 on HMGB1 localization and expression in the context of a differentiated epithelium we analyzed the expression of this protein in organotypic cultures expressing HPV16 oncogenes. We observed that HMGB1 exhibited a strong nuclear expression in cells from the basal layer, while gradually losing expression in more differentiated layers from rafts prepared with normal keratinocytes or keratinocytes expressing only HPV16 E6 (Fig. 3A,B). On the other hand, cultures expressing HPV16 E6E7 or HPV16 E7 exhibited strong nuclear HMGB1 signal in cells from all epithelial strata (Fig. 3C,D). We then analyzed HMGB1 expression in two cervical squamous cell carcinomas specimens (Fig. 3E–H). Similarly, to what was observed in organotypic cultures expressing HPV16 oncogenes, invasive tumor areas exhibited strong nuclear HMGB1 staining in all cells (Fig. 3F and H). On the other hand, strong HMGB1 signal was restricted to the basal cell layers of adjacent normal epithelium areas of the specimens (Fig. 3E and G).

### HMGB1 is important for the viability and proliferation of cervical cancer cell lines

**Figure 4:**
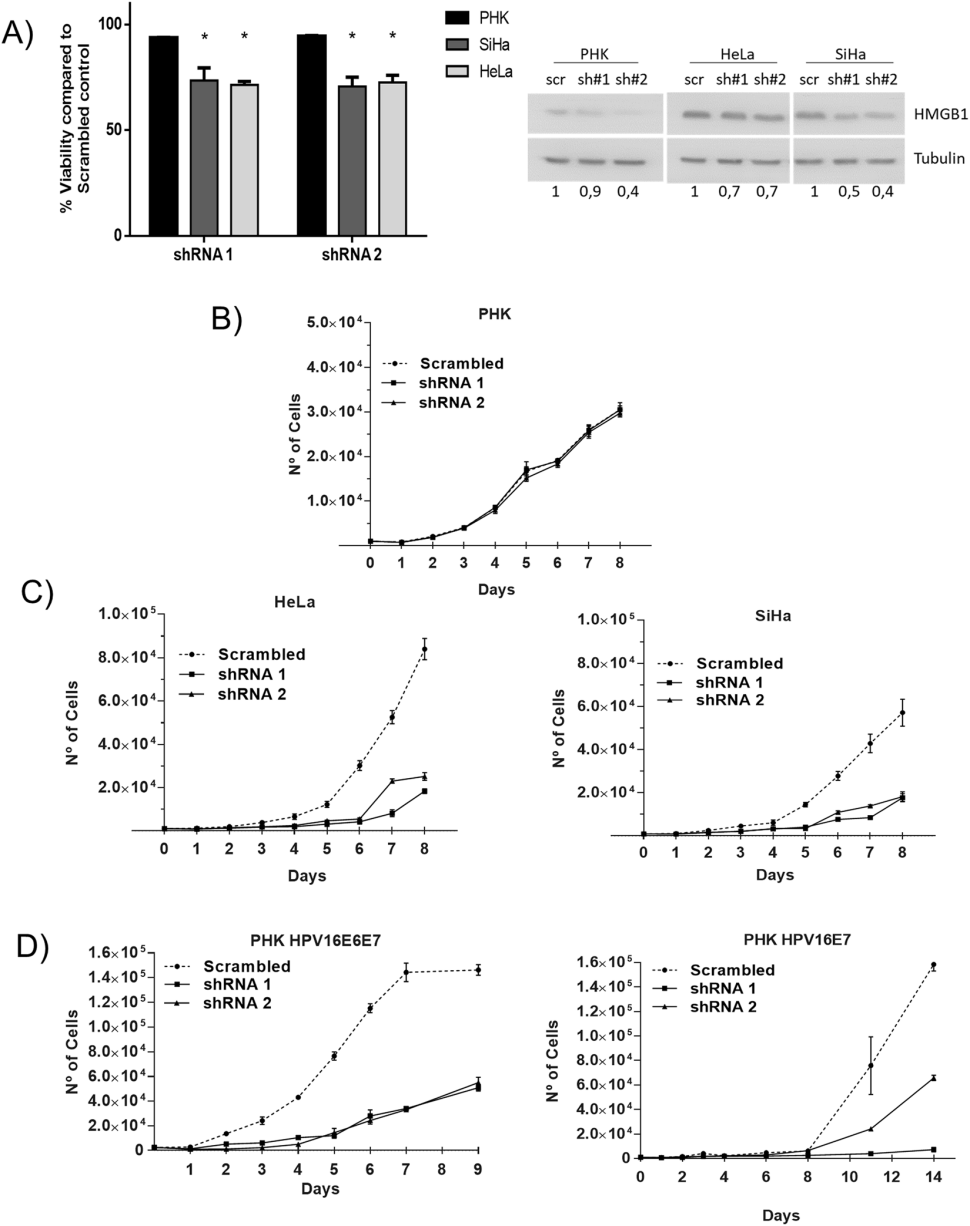
HMGB1 silencing selectively inhibits cell viability and proliferation of SiHa and HeLa cell lines. (**A**) Viability of silenced cell lines was determined by AlamarBlue reduction. Cells were seeded in 96-well plates (2000 cells/well) and infected with lentiviral particles expressing specific shRNAs. After five days, AlamarBlue was added (10 µl/well) and reagent reduction was determined by absorbance measurement after four hours. Viability inhibition values for each cell line are reported as relative to those observed in cells transduced with a scrambled shRNA. (**B**,**C**) HMGB1 silencing has no effect on normal keratinocytes proliferation (**B**), but inhibits SiHa and HeLa growth (**C**), as well as, PHK expressing HPV16 E6E7 or only E7 (**D**), as determined by proliferation curves. Cells previously transduced with lentivirus vectors for silencing HMGB1 were seeded (1000 cells/well in 24-well plates) and the number of cells was counted in triplicate for eight days. (Student’s *t* test, p ≤ 0.05).

Our results show that cervical cancer cell lines express high levels of HMGB1 (Fig. 2A). To gain insight into the biological relevance of this observation, we silenced HMGB1 in HeLa and SiHa cell lines using lentiviral-based specific shRNA. We observed that HMGB1 silencing resulted in reduced viability and proliferation of HeLa and SiHa cells (Fig. 4A–C), while normal PHK were not affected (Fig. 4A,B). Furthermore, PHK expressing HPV16 E7 alone or in combination with E6 were also sensitive to HMGB1 silencing (Fig. 4D). These results suggest that HMGB1 is required for the growth and survival of cells with HPV.

### HMGB1 silencing reduces the clonogenic potential of HPV-positive tumor cell lines

**Figure 5:**
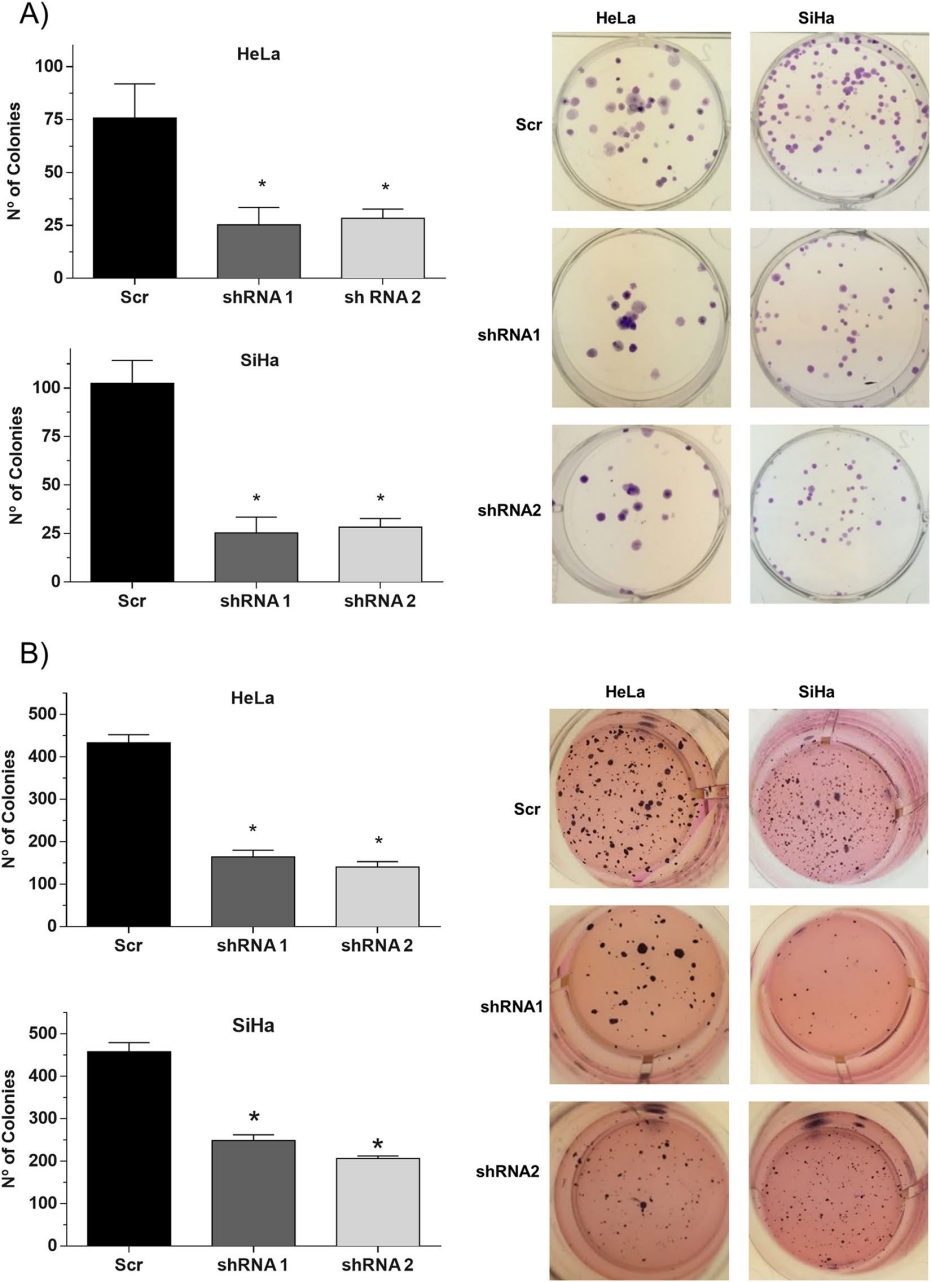
HMGB1 silencing affects the clonogenic potential of cervical cancer-derived cells. A lentiviral shRNA (MISSION®) was used specifically to silence HMGB1 in cervical cancer cell lines. After antibiotic selection, cells were seeded for colony formation assays. (**A**) Inhibition of colony formation in low-density assay. Cells were seeded in 6-well plates (100 cells/well); after 14 days, cells were fixed and the number of colonies was counted. (**B**) Inhibition of colony formation on soft agar in anchorage-independent growth assay. Cells were seeded in 24-well plates coated with agarose (500 cells/well). After 30 days, colonies were stained with MTT and counted. (Student’s *t* test, p ≤ 0.05).

Next, we tested if HMGB1 had an effect on the tumorigenic potential of HeLa and SiHa cells by determining the impact of HMGB1 silencing on the clonogenic potential of these cells. We observed that SiHa and HeLa cells exhibited reduced colony formation potential upon HMGB1 silencing (Fig. 5A). Besides, we analyzed the effect of HMGB1 silencing on the anchorage-independent growth potential of HPV-transformed cells using a soft agar assay. Once more, silencing HMGB1 resulted in a clear reduction of colonies formed in soft agar by treated HeLa and SiHa cells (Fig. 5B). Taken together, these results suggest that HMGB1 plays an important role in colony formation potential of cervical cancer cell lines.

## Discussion

Several studies have shown that signaling through TLR can contribute to tumor growth. TLR activation triggers the production of cytokines and chemokines that can promote angiogenesis, cell survival and chemoresistance, favoring tumor progression^12,13^.

Regulation of TLR-signaling is also relevant in the development of tumors associated with viral infections. For instance, some viruses such as human immunodeficiency virus (HIV-1), hepatitis C virus (HCV), and HPV16 cause persistent infection that often precedes tumor development. These viruses induce poor immune responses and lead to decreased expression and impaired TLR9 function^8,14–17^. In carcinomas associated with Merkel cell polyomavirus infection, virus presence also correlates with a strong decrease in TLR9 expression^18^.

Here we show that the expression of most TLRs is downregulated in cells transformed with HPV, the only exception being TLR4. Previous studies have shown a positive correlation between TLR4 expression levels and cervical intraepithelial neoplasia (CIN) grade^9,19^. In addition, activation of TLR4 by LPS in HPV positive cervical tumor derived cell lines resulted in higher TLR4 expression and resistance to apoptosis^19^. We also observed that other proteins related directly to TLR4 activation exhibited altered expression in the presence of HPV. These include the adaptor molecules MyD88 and SARM1, NFκB activation-related proteins Ube2N and TRAF6, and the TLR4 agonist HMGB1. Interestingly, while MyD88 was downregulated in cervical cancer cell lines, SARM1 expression levels were upregulated. The latter proteins have opposite effects on TLR signaling: MyD88 is responsible for TLR pathway activation, whereas SARM1 acts as a competitor for TLR binding and leads to signal disruption, inhibiting innate immune response triggered by TLR^20,21^. Taken together, these data indicate that not only inactivation of the TLR pathway may be beneficial for viral infection, but also the specific activation of certain components of the pathway may play an important role in viral tumorigenesis. Moreover, our observations suggest that SARM1 upregulation and MyD88 downregulation may be involved in HPV immune evasion.

Interestingly, in addition to its function in TLR-signaling regulation, increased SARM1 expression may play other roles in tumorigenesis. This protein participates in mitophagy together with TRAF6^22^ and can be translocated to the nucleus where it stabilizes lamins and protects them against degradation^23^. Additionally, SARM1 can lead to cell death after production of reactive oxygen species during oxidative stress, in a type of programmed cell death termed sarmoptosis^24^. Considering proteins downstream to TLR activation, Ube2N has been described as important for normal keratinocytes growth and survival^25^, while TRAF6 ubiquitination was previously reported to be increased in the presence of HPV18 E2 protein^26^. Besides, IRAK4 and other IRAK members are currently studied as potential therapeutic targets to a range of tumors^27^. Nevertheless, to the best of our knowledge, this is the first time that alterations in SARM1, Ube2N, TRAF6 and IRAK4 have been associated with HPV oncogene expression.

Gene expression analysis results showed that HMGB1 is upregulated in cervical cancer cell lines. HMGB1 is a relevant protein in DNA repair and chromatin organization that can be secreted from cells and regulate inflammation through TLR4 activation^10^. Several studies suggest a role of HMGB1 in enhancing expression and activation of topoisomerase II alpha in pRb-deficient tumor cells^28^. Moreover, pRb has been identified as a possible HMGB1 interaction partner^29^. Considering the high-risk HPV E7 oncoprotein targets pRb for degradation, it is plausible that HMGB1 may play a role in HPV-mediated cell transformation.

The expression of HMGB1 has been investigated in several tumor models. In lung adenocarcinoma, high levels of HMGB1 correlated positively with tumor staging and were associated with poor survival^30^. Additionally, in a melanoma mouse model it was observed that HMGB1 release promoted M2 macrophage recruitment and IL10 expression and was associated with tumor growth and metastasis^31^. Production of IL8 is also stimulated by HMGB1 and is responsible for enhanced migration and angiogenesis in gastric cancer^32^ and is upregulated in invasive cervical cancers^33^. Moreover, higher HMGB1 levels were detected in serum samples of patients with ovarian cancer as compared to controls and patients with benign tumors^34^. Finally, a study conducted using hepatocellular carcinoma cell lines showed that HMGB1 silencing inhibited *in vitro* cell proliferation, migration, invasion and ultimately led to cell apoptosis^35^.

A strong relationship between HMGB1, TLR4, RAGE receptors, and tumorigeneses has also been reported. HMGB1 secretion leads to a higher growth and invasive potential of lung cancer cell lines dependent of RAGE and TLR4 signaling^36^. Furthermore, in human airway epithelial cells, HMGB1 was also capable of inducing epithelial-mesenchymal transition by triggering PI3K/AKT/GSKβ/β-catenin signaling pathway through RAGE activation^37^. In addition, there is a correlation between TLR4 membrane expression and p65 nuclear localization during the progression from normal tissue to precursor lesions, to squamous cell carcinoma in human epidermal tumors. Besides, higher HMGB1 levels were observed in lesions compared to normal tissue^38^. Moreover, TLR4 signaling triggered by HMGB1 is able to stimulate an inflammatory response leading to tumor development in a skin model of tumorigenesis^39^. This inflammatory response induced by TLR4 and HMGB1, can also promote angiogenesis and metastasis in melanoma models after UV exposure^40^. Various types of tumor-derived cell lines release HMGB1 in different conditions, such as glucose deprivation, leading to increased proliferation, migration and invasion of stromal cells involved in all stages of tumor progression through TLR4 activation^41^. Interestingly, autophagic cancer-associated fibroblasts secrete HMGB1 activating TLR4 in luminal breast cancer cells, enhancing their stemness and tumorigenicity, correlating TLR4 activation with poor prognosis and increased relapse rate^42^. The results presented here support the hypothesis that HMGB1 silencing reduces cell viability, proliferation, and colony formation capacity of cervical cancer cell lines.

HPV infection may interfere with TLR pathways at different levels, resulting in the impairment of their activation and function potentially leading to innate immune system evasion. Moreover, TLR pathway proteins such as TLR4, SARM1, and HMGB1 may play additional roles related to tumor development. In conclusion, TLR4-HMGB1 signaling axis is a relevant pathway for survival of HPV-positive tumor cells, which warrants further studies to evaluate its potential as a therapeutic target or tumor biomarker.

## Materials and Methods

### Retroviral vectors, monolayer cells cultures, organotypic cultures and cervical samples

Pooled primary foreskin human keratinocytes (PHK) (Lonza, Basel, Switzerland) were grown in KSFM medium (Thermo Fisher, Waltham, MA, USA) supplemented with recombinant epidermal growth factor (5 ng/ml) and bovine pituitary extract (50 mg/ml) at 37 °C and 5% CO_2_. The cell lines used in the present study, C33A (ATCC® HTB-31™), HeLa (CCL-2 ATCC™), SiHa (ATCC® HTB-35™) and HEK293T (#), were purchased from the American Type Culture Collection (ATCC). All cell lines were cultured in MEM culture medium supplemented with 10% fetal bovine serum (M10 medium). PHK cells were transduced with retroviral vectors encoding the E6 and E7 proteins of HPV. Recombinant pLXSN retroviruses vectors containing HPV-16 E6/E7 were kindly provided by Dr. Denise Galloway (Fred Hutchinson Cancer Research Center, Seattle, WA) and are described elsewhere^43^. After 24 h of transduction, the cells were selected with geneticin (Thermo Fisher, Massachusetts) for 2 days, and then used to seed the epithelial raft cultures. After 10 days at the medium-air interface raft cultures were harvested. Cervical tissue samples were obtained from State University of Campinas, Brazil, where informed consent was obtained from all participants. Ethical approval was granted by the Comitê de Ética em Pesquisa of State University of Campinas (#1.647.260), and all methods were performed in accordance with the relevant guidelines and regulations, with informed consent obtained from all participants.

### Immunohistochemistry

For immunohistochemistry analysis of organotypic cultures samples, 4 µm sections were incubated with anti-HMGB1 antibody (Abcam, Cambridge, MA, UK) at 1:1000 dilutions. Paraffin-embedded cervical tissue specimens of invasive squamous carcinomas were tested as well with the anti-HMGB1 antibody at 1:100 dilution. All immunohistochemistry were performed in a Ventana Benchmark GX equipment 234 (Ventana Medical Systems, Arizona, USA) according to the manufacturer’s instructions.

### RNA purification and PCR array

For each sample, total RNA was extracted using RNeasy Mini Kit columns (Qiagen, Hilden, Germany) according to the manufacturer’s instructions. Gene expression analysis was performed using the RNA Toll-Like Receptor Signaling Pathway PCR Array (Qiagen, Hilden, Germany) according to the manufacturer’s instructions. Briefly, 2 µg of total RNA was treated with DNAse for 5 min and reverse-transcribed. The resulting cDNA was mixed with a mastermix solution and specific primers and analyzed by quantitative PCR on a 7500 Real-Time PCR system (Applied Biosystems, Foster City, CA, USA). Analysis was performed using the RT² Profiler PCR Array Data Analysis online software (Qiagen, Hilden, Germany). A functional network was generated using String (http://string-db.org/). Samples were assayed in independent triplicates.

### Protein extraction and immunoblotting

Total protein extracts were obtained after lysing cells grown in monolayer in lysis buffer containing 150 mM NaCl, 5 mM EDTA, 50 mM Tris-HCI pH 8.0, 1% NP-40, 0.5% Na-deoxycholate, 0.1% SDS and supplemented with protease inhibitors. Protein concentration was determined by the Bradford method using the Bio-Rad protein assay (Bio-Rad, Hercules, CA, USA). Primary antibodies used included anti-HMGB1 antibody [EPR3507] (ab79823), anti-Ube2N/Ubc13 antibody [EPR5162] (ab109286), anti-MyD88 antibody (ab2064), anti-TRAF6 antibody [EP591Y] (ab33915), anti-TLR4 antibody [76B357.1] (ab22048), and anti-SARM antibody (ab115561). All antibodies were purchased from Abcam (Cambridge, United Kingdom). The following secondary antibodies were used: anti-mouse (115-035-003) or anti-rabbit (111-035-003) (Jackson ImmunoResearch, West Grove, PA, USA). Peroxidase activity from secondary antibodies was assessed using the ECL kit (Amersham Biosciences, GE Healthcare, Chicago, IL, USA). Signals were quantified using ImageJ software (National Institutes of Health, Maryland, USA).

### Lentivirus production and gene silencing

HMGB1 silencing was performed using lentiviral vectors expressing specific shRNAs (NM_002128.3-302s1c1; NM_002128.3-188s1c1) or a scrambled sequence (MISSION® Lentiviral System; Sigma-Aldrich, St. Louis, MO, USA). Lentiviral particles were produced in HEK293T cells according to the manufacturer’s instructions. Virus-containing supernatant was filtered and used to transduce SiHa, HeLa, and PHK cells. After 48 hours, cells were selected using medium added with 2.5 μg/ml of puromycin (Thermo Fisher, Massachusetts, USA).

### Cell viability assay

For cell viability analysis, cells (2000 cells/well) were seeded in 96 well plates and transduced with lentiviral particles expressing shRNA targeting HMGB1 or a scrambled sequence to serve as a control (MOI = 10). After five days, AlamarBlue reagent (Thermo Fisher, Massachusetts, USA) was added to the cells at 10% final concentration (v:v), according to the manufacturer’s instructions. Cells were incubated at 37 °C for 4 hours. After this point, Alamar Blue’s reduction was monitored at 570 and 600 nm at 1 h intervals for 8 h, in an Epoch Microplate Spectrophotometer (Bio-Tek, Winooski, VT, USA).

### Clonogenic and proliferation assays

HeLa and SiHa cells previously transduced with lentiviral particles expressing HMGB1 and control shRNAs and selected with antibiotics, were seeded in triplicate. For the low-density clonogenic assay, 100 cells/well were plated in 6-well plates. After 14 days, colonies were fixed with 70% ethanol and stained with 0.5% crystal violet in 10% ethanol. For the anchorage-independent growth assay, five-hundred HeLa and SiHa cells were suspended in 500 µl of M10 with 0.6% agarose and plated in a 24-well plate previously coated with 1 ml of 1% agarose. After 30 days, colonies were stained with MTT (3-(4,5-dimethylthiazol-2-yl)-2,5-diphenyltetrazolium bromide) and counted. For cell growth curve, 1000 HeLa and SiHa cells were seeded per well in 24-well plates and counted once daily for eight days. For each assay, three independent experiments were performed in triplicate.

### Statistical analysis

Results were analyzed using the Student’s *t* test and analysis was performed on GraphPad Prism (GraphPad Software Inc., La Jolla, CA, USA). A P value < 0.05 was considered significant and indicated with asterisks.

### Data availability

All data generated or analyzed during this study are included in this published article (and its Supplementary Information files).

## Electronic supplementary material

Complete blots 1

Complete blots 2

Complete blots 2
