# Supplementary figures and images for: HPV-transformed cells exhibit altered HMGB1-TLR4/MyD88-SARM1 signaling axis
Source: Sci Rep. 2018 Feb 22;8:3476. doi: 10.1038/s41598-018-21416-8 (PMC5823898; doi:10.1038/s41598-018-21416-8)

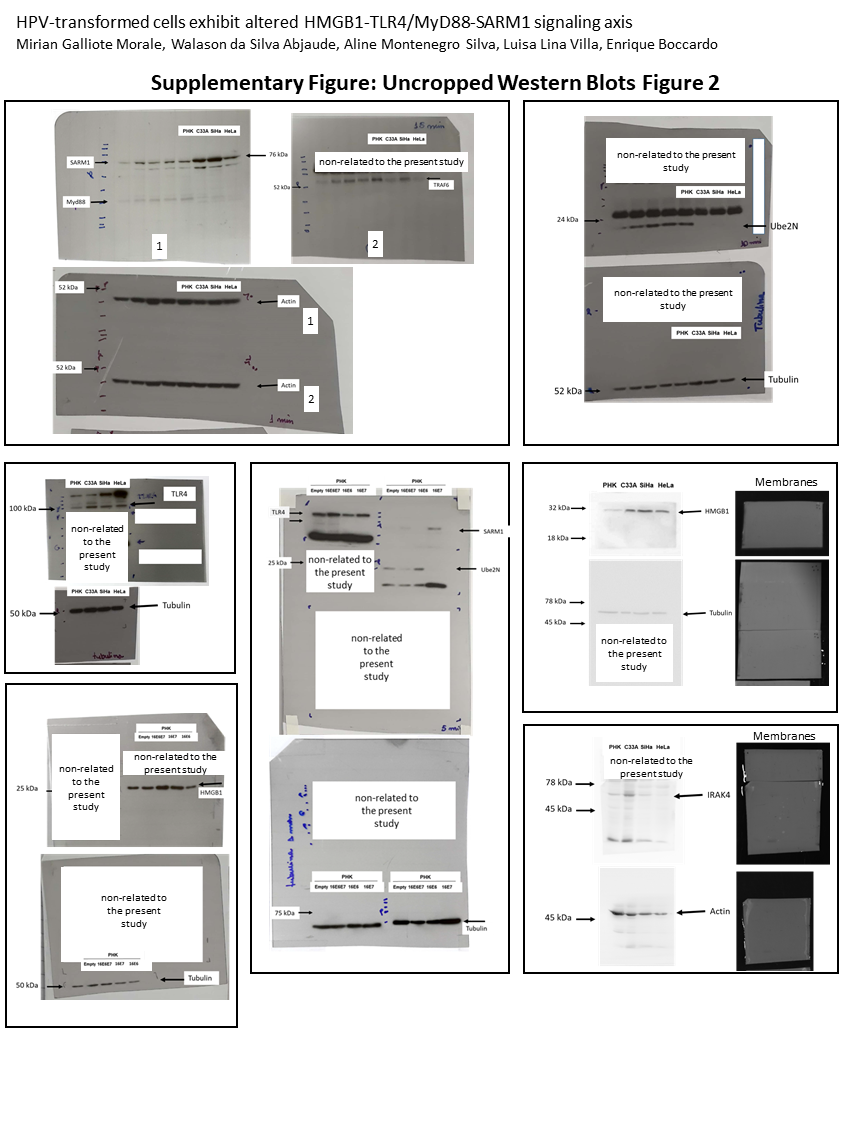

Supplement: Supplementary file 1 — Complete blots 1 [file 41598_2018_21416_MOESM1_ESM.tif]

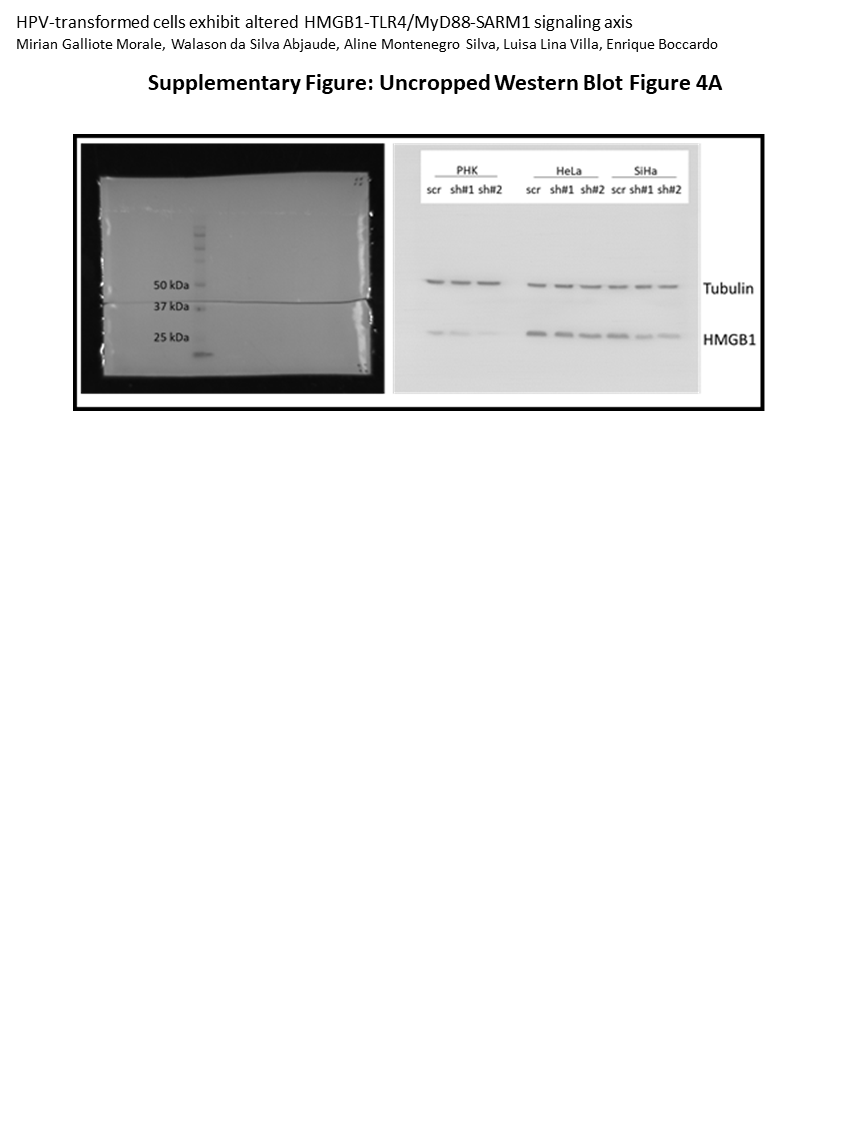

Supplement: Supplementary file 2 — Complete blots 2 [file 41598_2018_21416_MOESM2_ESM.tif]
