# Supplementary material for: HPV-transformed cells exhibit altered HMGB1-TLR4/MyD88-SARM1 signaling axis
Source: Sci Rep. 2018 Feb 22;8:3476. doi: 10.1038/s41598-018-21416-8 (PMC5823898; doi:10.1038/s41598-018-21416-8)
Supplement: Supplementary file 3 — Complete blots 2 [file 41598_2018_21416_MOESM3_ESM.pdf]

HPV-transformed cells exhibit altered HMGB1-TLR4/MyD88-SARM1 signaling axis

Mirian Galliote Morale, Walason da Silva Abjaude, Aline Montenegro Silva, Luisa Lina Villa, Enrique Boccardo

PCR Array Catalog #:		PAHS-018Z

Position	Unigene	Refseq	Symbol	Description	Gname	RT2 Catalog
A01	Hs.159494	NM_000061	BTK	Bruton agammaglobulinemia tyrosine kinase	AGMX1/AT/ATK/BPK/IN	PPH00086A
A02	Hs.599762	NM_001228	CASP8	Caspase 8, apoptosis-related cysteine peptidase	ALPS2B/CAP4/Casp-8/F	PPH00359F
A03	Hs.303649	NM_002982	CCL2	Chemokine (C-C motif) ligand 2	GDCF-2/HC11/HSMCR3	PPH00192F
A04	Hs.163867	NM_000591	CD14	CD14 molecule	-	PPH05723A
A05	Hs.87205	NM_005582	CD180	CD180 molecule	LY64/Ly78/MGC126233	PPH06054A
A06	Hs.838	NM_005191	CD80	CD80 molecule	B7/B7-1/B7.1/BB1/CD2	PPH00860F
A07	Hs.171182	NM_006889	CD86	CD86 molecule	B7-2/B7.2/B70/CD28LG	PPH00826A
A08	Hs.198998	NM_001278	CHUK	Conserved helix-loop-helix ubiquitous kinase	IKBKA/IKK-alpha/IKK1/I	PPH00649C
A09	Hs.236516	NM_014358	CLEC4E	C-type lectin domain family 4, member E	CLECSF9/MINCLE	PPH06040A
A10	Hs.1349	NM_000758	CSF2	Colony stimulating factor 2 (granulocyte-macrophag	GMCSF/MGC131935/M	PPH00576C
A11	Hs.2233	NM_000759	CSF3	Colony stimulating factor 3 (granulocyte)	C17orf33/CSF3OS/GCSF	PPH00723B
A12	Hs.632586	NM_001565	CXCL10	Chemokine (C-X-C motif) ligand 10	C7/IFI10/INP10/IP-10/S	PPH00765E
B01	Hs.515146	NM_016581	ECSIT	ECSIT homolog (Drosophila)	SITPEC	PPH06039A
B02	Hs.131431	NM_002759	EIF2AK2	Eukaryotic translation initiation factor 2-alpha kinas	EIF2AK1/MGC126524/F	PPH01327B
B03	Hs.181128	NM_005229	ELK1	ELK1, member of ETS oncogene family	-	PPH00140C
B04	Hs.86131	NM_003824	FADD	Fas (TNFRSF6)-associated via death domain	MGC8528/MORT1	PPH00367A
B05	Hs.728789	NM_005252	FOS	FBJ murine osteosarcoma viral oncogene homolog	AP-1/C-FOS	PPH00094A
B06	Hs.593339	NM_002128	HMGB1	High mobility group box 1	DKFZp686A04236/HMG	PPH00999F
B07	Hs.37003	NM_005343	HRAS	V-Ha-ras Harvey rat sarcoma viral oncogene homolo	C-BAS/HAS/C-H-RAS/C-	PPH00159C
B08	Hs.728810	NM_005345	HSPA1A	Heat shock 70kDa protein 1A	FLJ54303/FLJ54370/FLJ	PPH01193B
B09	Hs.595053	NM_002156	HSPD1	Heat shock 60kDa protein 1 (chaperonin)	CPN60/GROEL/HLD4/H	PPH01205A
B10	Hs.37026	NM_024013	IFNA1	Interferon, alpha 1	IFL/IFN/IFN-ALPHA/IFN	PPH01321B
B11	Hs.93177	NM_002176	IFNB1	Interferon, beta 1, fibroblast	IFB/IFF/IFNB/MGC9695	PPH00384F
B12	Hs.856	NM_000619	IFNG	Interferon, gamma	IFG/IFI	PPH00380C
C01	Hs.597664	NM_001556	IKBKB	Inhibitor of kappa light polypeptide gene enhancer	FLJ33771/FLJ36218/FLJ	PPH00780C
C02	Hs.193717	NM_000572	IL10	Interleukin 10	CSIF/IL-10/IL10A/MGC1	PPH00572C
C03	Hs.673	NM_000882	IL12A	Interleukin 12A (natural killer cell stimulatory factor	CLMF/IL-12A/NFSK/NKS	PPH00544B
C04	Hs.1722	NM_000575	IL1A	Interleukin 1, alpha	IL-1A/IL1/IL1-ALPHA/IL	PPH00690A
C05	Hs.126256	NM_000576	IL1B	Interleukin 1, beta	IL-1/IL1-BETA/IL1F2	PPH00171C
C06	Hs.89679	NM_000586	IL2	Interleukin 2	IL-2/TCGF/lymphokine	PPH00172C
C07	Hs.654458	NM_000600	IL6	Interleukin 6 (interferon, beta 2)	BSF2/HGF/HSF/IFNB2/I	PPH00560C
C08	Hs.624	NM_000584	IL8	Interleukin 8	CXCL8/GCP-1/GCP1/LE	PPH00568A
C09	Hs.522819	NM_001569	IRAK1	Interleukin-1 receptor-associated kinase 1	IRAK/pelle	PPH00835A
C10	Hs.449207	NM_001570	IRAK2	Interleukin-1 receptor-associated kinase 2	IRAK-2/MGC150550	PPH01800C
C11	Hs.138499	NM_016123	IRAK4	Interleukin-1 receptor-associated kinase 4	IPD1/NY-REN-64/REN6	PPH06036A
C12	Hs.436061	NM_002198	IRF1	Interferon regulatory factor 1	IRF-1/MAR	PPH00320F
D01	Hs.75254	NM_001571	IRF3	Interferon regulatory factor 3	-	PPH02025B
D02	Hs.714791	NM_002228	JUN	Jun proto-oncogene	AP-1/AP1/c-Jun	PPH00095A
D03	Hs.36	NM_000595	LTA	Lymphotoxin alpha (TNF superfamily, member 1)	LT/TNFB/TNFSF1	PPH00337F
D04	Hs.653138	NM_004271	LY86	Lymphocyte antigen 86	MD-1/MMD-1/dJ80N2.	PPH06038F
D05	Hs.660766	NM_015364	LY96	Lymphocyte antigen 96	ESOP-1/MD-2/MD2/ly-	PPH06052A
D06	Hs.514012	NM_002756	MAP2K3	Mitogen-activated protein kinase kinase 3	MAPKK3/MEK3/MKK3/	PPH00747F
D07	Hs.514681	NM_003010	MAP2K4	Mitogen-activated protein kinase kinase 4	JNKK/JNKK1/MAPKK4/M	PPH00195C
D08	Hs.657756	NM_005921	MAP3K1	Mitogen-activated protein kinase kinase kinase 1	MAPKKK1/MEKK/MEKK	PPH00706C
D09	Hs.644143	NM_003188	MAP3K7	Mitogen-activated protein kinase kinase kinase 7	MEKK7/TAK1/TGF1a	PPH00749C
D10	Hs.431550	NM_004834	MAP4K4	Mitogen-activated protein kinase kinase kinase kina	FLH21957/FLJ10410/FL	PPH06047A
D11	Hs.138211	NM_002750	MAPK8	Mitogen-activated protein kinase 8	JNK/JNK1/JNK1A2/JNK2	PPH00720B
D12	Hs.207763	NM_015133	MAPK8IP3	Mitogen-activated protein kinase 8 interacting prot	DKFZp762N1113/FLJ00	PPH06051A
E01	Hs.82116	NM_002468	MYD88	Myeloid differentiation primary response gene (88)	MYD88D	PPH00911B
E02	Hs.654408	NM_003998	NFKB1	Nuclear factor of kappa light polypeptide gene enha	DKFZp686C01211/EBP-	PPH00204F
E03	Hs.73090	NM_002502	NFKB2	Nuclear factor of kappa light polypeptide gene enha	LYT-10/LYT10/p52	PPH00782F
E04	Hs.81328	NM_020529	NFKBIA	Nuclear factor of kappa light polypeptide gene enha	IKBA/MAD-3/NFKBI	PPH00170F
E05	Hs.2764	NM_005007	NFKBIL1	Nuclear factor of kappa light polypeptide gene enha	IKBL/LST1/NFKBIL	PPH01802A
E06	Hs.530539	NM_006165	NFRKB	Nuclear factor related to kappaB binding protein	DKFZp547B2013/INO80	PPH01997B
E07	Hs.591667	NM_003298	NR2C2	Nuclear receptor subfamily 2, group C, member 2	TAK1/TR2R1/TR4/hTAK	PPH01268F
E08	Hs.7886	NM_020651	PELI1	Pellino homolog 1 (Drosophila)	DKFZp686C18116/MGC	PPH06053A
E09	Hs.103110	NM_005036	PPARA	Peroxisome proliferator-activated receptor alpha	MGC2237/MGC2452/N	PPH01281B
E10	Hs.570274	NM_003690	PRKRA	Protein kinase, interferon-inducible double strande	DYT16/PACT/RAX	PPH02171F
E11	Hs.196384	NM_000963	PTGS2	Prostaglandin-endoperoxide synthase 2 (prostaglan	COX-2/COX2/GRIPGHS/	PPH01136F
E12	Hs.631886	NM_002908	REL	V-rel reticuloendotheliosis viral oncogene homolog	C-Rel	PPH00101B
F01	Hs.502875	NM_021975	RELA	V-rel reticuloendotheliosis viral oncogene homolog	MGC131774/NFKB3/p6	PPH01812B
F02	Hs.103755	NM_003821	RIPK2	Receptor-interacting serine-threonine kinase 2	CARD3/CARDIAK/CCK/	PPH00881C
F03	Hs.532781	NM_015077	SARM1	Sterile alpha and TIR motif containing 1	FLJ36296/KIAA0524/SA	PPH06037A
F04	Hs.501624	NM_021805	SIGIRR	Single immunoglobulin and toll-interleukin 1 recept	MGC110992/TIR8	PPH06049A
F05	Hs.507681	NM_006116	TAB1	TGF-beta activated kinase 1/MAP3K7 binding prote	3'-Tab1/MAP3K7IP1/M	PPH01804B
F06	Hs.505874	NM_013254	TBK1	TANK-binding kinase 1	FLJ11330/NAK/T2K	PPH01797E
F07	Hs.29344	NM_182919	TICAM1	Toll-like receptor adaptor molecule 1	MGC35334/PRVTIRB/TI	PPH06044A
F08	Hs.710895	NM_021649	TICAM2	Toll-like receptor adaptor molecule 2	MGC129876/MGC1298	PPH06042A
F09	Hs.537126	NM_001039661	TIRAP	Toll-interleukin 1 receptor (TIR) domain containing	FLJ42305/Mal/wyatt	PPH06246B
F10	Hs.654532	NM_003263	TLR1	Toll-like receptor 1	CD281/DKFZp547I0610	PPH01799A
F11	Hs.120551	NM_030956	TLR10	Toll-like receptor 10	CD290/MGC104967/M	PPH01794B
F12	Hs.519033	NM_003264	TLR2	Toll-like receptor 2	CD282/TIL4	PPH01808A
G01	Hs.657724	NM_003265	TLR3	Toll-like receptor 3	CD283	PPH01803E
G02	Hs.174312	NM_138554	TLR4	Toll-like receptor 4	ARMD10/CD284/TOLL/	PPH01795F
G03	Hs.604542	NM_003268	TLR5	Toll-like receptor 5	FLJ10052/MGC126430/	PPH01793F
G04	Hs.662185	NM_006068	TLR6	Toll-like receptor 6	CD286	PPH01798E
G05	Hs.659215	NM_016562	TLR7	Toll-like receptor 7	-	PPH01796A
G06	Hs.660543	NM_138636	TLR8	Toll-like receptor 8	CD288/MGC119599/M	PPH01801B
G07	Hs.87968	NM_017442	TLR9	Toll-like receptor 9	CD289	PPH01809A
G08	Hs.241570	NM_000594	TNF	Tumor necrosis factor	DIF/TNF-alpha/TNFA/TI	PPH00341F
G09	Hs.279594	NM_001065	TNFRSF1A	Tumor necrosis factor receptor superfamily, membe	CD120a/FPF/MGC1958	PPH00346C
G10	Hs.368527	NM_019009	TOLLIP	Toll interacting protein	FLJ33531/IL-1RAcPIP	PPH05844C
G11	Hs.591983	NM_004620	TRAF6	TNF receptor-associated factor 6	MGC:3310/RNF85	PPH00329B
G12	Hs.524630	NM_003348	UBE2N	Ubiquitin-conjugating enzyme E2N	MGC131857/MGC8489	PPH02724A

HPV-transformed cells exhibit altered HMGB1-TLR4/MyD88-SARM1 si

Mirian Galliote Morale, Walason da Silva Abjaude, Aline Montenegro Silva, Luisa Lina

Supplementary Table I

	Relative to control group PHK					
	C33A		HeLa		SiHa	
Symbol	Fold Regulation	pValue	Fold Regulation	pValue	Fold Regulation	pValue
BTK	-2,0127	0,26774	-2,682	0,31192	-1,9767	0,26601
CASP8	-83,2378	0,000119	-1,879	0,004909	-1,293	0,28434
CCL2	-8,1257	0,08706	16,374	0,000419	-4,016	0,12958
CD14	-2,5713	0,21728	1,1975	0,78418	167,6511	0,05265
CD180	1,2694	0,1531	6,4086	0,045922	-1,2941	0,39898
CD80	1,2991	0,86404	-5,2416	0,23214	-2,6905	0,26373
CD86	-34,2769	0,24308	-550,0174	0,23724	-293,0857	0,23762
CHUK	1,7023	0,036454	-1,674	0,36163	-1,2886	0,482
CLEC4E	2,7085	0,045802	-5,9244	0,07711	-3,1569	0,1215
CSF2	-13,3846	0,15123	-28,3118	0,14384	-16,8316	0,14754
CSF3	-530,975	0,24474	-3911,0312	0,24439	-223,4831	0,24573
CXCL10	-49,3793	0,27322	-348,8992	0,27076	-32,4396	0,27476
ECSIT	1,4582	0,65998	1,5122	0,58316	1,5687	0,53848
EIF2AK2	1,0335	0,91389	-7,8354	0,029994	-1,9179	0,15595
ELK1	1,1388	0,88879	2,9214	0,11508	6,6822	0,05454
FADD	-2,3174	0,33356	3,1239	0,24655	3,3971	0,17962
FOS	-12,5461	0,20713	-42,7149	0,19298	-3,8755	0,26085
HMGB1	5,5053	0,003132	-3,302	0,035422	-1,1123	0,55468
HRAS	-1,6731	0,40946	1	0,61422	2,0233	0,7379
HSPA1A	1,4381	0,4007	6,1333	0,07625	13,0453	0,06569
HSPD1	2,8366	0,001375	2,969	0,038492	1,7514	0,01244
IFNA1	-8,3349	0,017002	-2,4509	0,06449	-9,9106	0,016054
IFNB1	1,0169	0,78102	-11,4188	0,09667	-2,1063	0,23465
IFNG	1,3112	0,86011	-7,6919	0,23894	-6,6277	0,23178
IKBKB	1,4348	0,041726	-2,6512	0,005166	-1,7616	0,11814
IL10	1,1102	0,91086	-4,7131	0,17081	-2,1978	0,25463
IL12A	3,7257	0,00008	-5,2054	0,001139	-2,6503	0,001863
IL1A	-2725,868	0,002619	-820,2956	0,002627	-478,054	0,002635
IL1B	-2584,7935	0,005522	-2407,5221	0,005522	-187,3754	0,005618
IL2	1,9919	0,06059	-4,122	0,10419	-3,479	0,05668
IL6	-22,9831	0,08949	2,8284	0,05171	20,5483	0,11835
IL8	-35,0781	0,043832	-9,4698	0,05516	-2,6102	0,20035
IRAK1	-1,2079	0,51816	1,923	0,40738	-1,2899	0,767
IRAK2	-16,1392	0,20829	-4,801	0,24909	-2,5066	0,31885
IRAK4	4,0395	0,000162	-5,8971	0,009925	-2,9904	0,046068
IRF1	-1,8308	0,31468	-2,3674	0,24899	1,7976	0,42603
IRF3	2,804	0,000191	-4,2871	0,004676	1,0351	0,87973
JUN	-10,6233	0,32423	1,4044	0,60064	1,1673	0,55342
LTA	-1,2447	0,27194	1,1225	0,67922	1,6716	0,72184
LY86	-1,183	0,53499	-4,4485	0,23516	-1,8697	0,30594
LY96	-18,885	0,038116	-739,2917	0,032008	-99,8918	0,032759
MAP2K3	2,3201	0,11971	-4,3873	0,11201	-2,2902	0,17686
MAP2K4	2,1798	0,000969	-3,4742	0,006684	-1,3254	0,32402
MAP3K1	-1,3972	0,014551	-2,8219	0,000499	-1,1017	0,62034
MAP3K7	1,8245	0,001959	-3,1821	0,004372	-2,1965	0,005102
MAP4K4	-1,7441	0,033613	-4,9019	0,005128	-2,7319	0,009046
MAPK8	3,1913	0,002086	-4,1602	0,001779	-1,4706	0,11744
MAPK8IP3	-1,1968	0,60634	-1,0234	0,67173	2,8174	0,25313
MYD88	-28,2955	0,036589	-7,1437	0,05029	-3,2794	0,07961
NFKB1	-1,5755	0,38623	-2,4509	0,25439	1,4911	0,74307
NFKB2	-1,1534	0,57019	-3,1675	0,1334	1,1676	0,95298
NFKBIA	-4,2945	0,05407	-3,8459	0,06086	1,7088	0,19092
NFKBIL1	-1,1375	0,25913	314,4456	0,37317	11,3665	0,37922
NFRKB	2,8366	0,000473	-2,1435	0,014417	1,0564	0,74442
NR2C2	1,1154	0,59389	-1,2058	0,23761	-1,1187	0,67809
PELI1	-2,9673	0,013267	-10,9536	0,003752	-6,1177	0,00509
PPARA	-1,2276	0,42583	-2,0801	0,11031	-1,1911	0,5647
PRKRA	1,8457	0,000619	-2,3729	0,017085	-1,4253	0,05395
PTGS2	-1356,6504	0,000401	-174,047	0,00041	-19,0958	0,000579
REL	1,0625	0,69082	-4,2871	0,000901	-2,9885	0,001503
RELA	1,3325	0,19038	-1,4607	0,13816	-1,0122	0,99174
RIPK2	2,441	0,00028	-5,439	0,002107	-2,5779	0,004401
SARM1	18,2628	0,000167	6,9483	0,003589	116,056	0,021559
SIGIRR	-4,4976	0,12346	-4,7131	0,15228	-2,9575	0,15776
TAB1	1,3763	0,74159	1,0994	0,9056	1,7426	0,417
TBK1	5,1844	0,012517	-2,2038	0,10235	1,1418	0,77775
TICAM1	-1,7603	0,027445	-5,0397	0,001533	-5,6368	0,001282
TICAM2	-1,9086	0,07942	-3,4105	0,032758	-2,0449	0,07373
TIRAP	1,3605	0,98724	-1,2002	0,52488	2,4155	0,343
TLR1	-1,9988	0,15861	-1,1329	0,65095	-16,3299	0,043127
TLR10	2,413	0,06974	-8,6939	0,002249	-4,741	0,004898
TLR2	-77,1271	0,006191	-560,2784	0,005957	-55,5681	0,006316
TLR3	-2,1079	0,025469	-12,4092	0,001999	-4,4118	0,00518
TLR4	-1,4871	0,26921	38,4968	0,000309	8,8637	0,038814
TLR5	-3,1077	0,045124	-27,5376	0,011405	-11,9529	0,012987
TLR6	-7,8671	0,05446	1,8575	0,1841	-6,0691	0,06002
TLR7	1,6142	0,76684	-5,5404	0,23666	-2,1057	0,32275
TLR8	2,7085	0,045802	-5,9244	0,07711	-1,6863	0,28469
TLR9	-1,0564	0,66105	-5,3025	0,046891	-2,9767	0,08963
TNF	-30,1169	0,11309	-103,9683	0,10839	-257,5149	0,10721
TNFRSF1	-78,7477	0,25122	2,4794	0,64225	4,24	0,18183
TOLLIP	-2,2436	0,33594	-4,1125	0,26481	1,3934	0,99369
TRAF6	1,1817	0,72474	-6,0071	0,028971	-3,0218	0,05203
UBE2N	2,8962	0,001254	-3,2117	0,022873	-1,8502	0,06162

HPV-transformed cells exhibit altered HMGB1-TL

Mirian Galliote Morale, Walason da Silva Abjaude, Aline Mor

Suppementary Table II

	Relative to control group C33A			
	HeLa		SiHa	
Symbol	Fold Regulation	pvalue	Fold Regulation	pvalue
BTK	-1,3325	0,82536	1,0182	0,97566
CASP8	44,2979	0,000459	64,3754	0,006568
CCL2	133,0508	0,00023	2,0234	0,25882
CD14	3,0791	0,02003	431,0819	0,05185
CD180	5,0484	0,05275	-1,6428	0,10497
CD80	-6,8093	0,08955	-3,4952	0,09671
CD86	-16,0463	0,000782	-8,5505	0,001185
CHUK	-2,8497	0,020659	-2,1936	0,012108
CLEC4E	-16,0463	0,000782	-8,5505	0,001185
CSF2	-2,1153	0,17055	-1,2575	0,044947
CSF3	-7,3658	0,000752	2,3759	0,29205
CXCL10	-7,0657	0,007418	1,5222	0,016674
ECSIT	1,0371	0,76381	1,0758	0,49947
EIF2AK2	-8,0976	0,00013	-1,9821	0,015062
ELK1	2,5654	0,01603	5,8678	0,04476
FADD	7,2392	0,014137	7,8725	0,005284
FOS	-3,4046	0,014472	3,2373	0,020271
HMGB1	-18,1786	0,001462	-6,1237	0,002273
HRAS	1,6731	0,045665	3,3851	0,000884
HSPA1A	4,2649	0,08935	9,0712	0,07057
HSPD1	1,0467	0,73592	-1,6196	0,005993
IFNA1	3,4007	0,016535	-1,189	0,29627
IFNB1	-11,6116	0,000872	-2,1419	0,006189
IFNG	-10,0852	0,003523	-8,6899	0,001893
IKBKB	-3,804	0,000288	-2,5275	0,012053
IL10	-5,2325	0,014727	-2,4401	0,024059
IL12A	-19,3935	0,000024	-9,8741	0,000025
IL1A	3,323	0,019347	5,702	0,000396
IL1B	1,0736	0,87351	13,7947	0,000428
IL2	-8,2107	0,004273	-6,9299	0,001227
IL6	65,006	0,00132	472,2626	0,10734
IL8	3,7042	0,14185	13,4387	0,12697
IRAK1	2,3227	0,1142	-1,0679	0,74533
IRAK2	3,3616	0,011928	6,4388	0,015567
IRAK4	-23,8211	0,000005	-12,0795	0,000032
IRF1	-1,2931	0,06958	3,2911	0,028877
IRF3	-12,0211	0,000002	-2,7089	0,000032
JUN	14,9199	0,000123	12,4003	0,011237
LTA	1,3972	0,44677	2,0807	0,10251
LY86	-3,7603	0,10947	-1,5804	0,07227
LY96	-39,1471	0,05333	-5,2895	0,08006
MAP2K3	-10,1788	0,002001	-5,3134	0,002747
MAP2K4	-7,5728	0,000007	-2,889	0,001077
MAP3K1	-2,0197	0,001634	1,2682	0,20896
MAP3K7	-5,8058	0,000113	-4,0074	0,000059
MAP4K4	-2,8105	0,003071	-1,5663	0,003631
MAPK8	-13,2768	0,000535	-4,6931	0,001351
MAPK8IP3	1,1695	0,74705	3,3718	0,14613
MYD88	3,9609	0,024791	8,6284	0,000081
NFKB1	-1,5556	0,021443	2,3493	0,011567
NFKB2	-2,7463	0,007176	1,3466	0,12899
NFKBIA	1,1166	0,45065	7,3385	0,004164
NFKBIL1	357,6736	0,37311	12,9291	0,36726
NFRKB	-6,0804	0,000128	-2,6852	0,002499
NR2C2	-1,3449	0,1824	-1,2477	0,42706
PELI1	-3,6914	0,007023	-2,0617	0,024413
PPARA	-1,6945	0,036971	1,0306	0,79836
PRKRA	-4,3797	0,000305	-2,6307	0,000134
PTGS2	7,7947	0,019438	71,0444	0,06862
REL	-4,5552	0,006134	-3,1753	0,00952
RELA	-1,9464	0,001783	-1,3488	0,23024
RIPK2	-13,2768	0,000007	-6,2927	0,000005
SARM1	-2,6284	0,000573	6,3548	0,034308
SIGIRR	-1,0479	0,74389	1,5207	0,29629
TAB1	-1,2519	0,22371	1,2661	0,42173
TBK1	-11,4254	0,007294	-4,5406	0,012039
TICAM1	-2,8629	0,008868	-3,2022	0,006375
TICAM2	-1,787	0,025878	-1,0714	0,63927
TIRAP	-1,633	0,002691	1,7754	0,27499
TLR1	1,7644	0,1509	-8,1697	0,000542
TLR10	-20,9784	0,016579	-11,44	0,019495
TLR2	-7,2643	0,004585	1,388	0,24966
TLR3	-5,8869	0,006384	-2,0929	0,05571
TLR4	57,2485	0,000185	13,1812	0,010956
TLR5	-8,8612	0,029067	-3,8462	0,042195
TLR6	14,6129	0,004008	1,2963	0,29057
TLR7	-8,9435	0,006584	-3,3991	0,007503
TLR8	-16,0463	0,000782	-4,5672	0,003118
TLR9	-5,0193	0,003938	-2,8177	0,019519
TNF	-3,4522	0,004907	-8,5505	0,001185
TNFRSF1	195,2484	0,002258	333,888	0,006062
TOLLIP	-1,8329	0,002747	3,1262	0,09317
TRAF6	-7,0984	0,000037	-3,5707	0,000087
UBE2N	-9,3017	0,000061	-5,3587	0,000068
